# Evaluation of Biological Activities and Medicinal Properties of Honey Drops and Honey Lozenges

**DOI:** 10.3390/nu14224738

**Published:** 2022-11-10

**Authors:** Petra Larsen, Marya Ahmed

**Affiliations:** 1 Department of Chemistry, University of Prince Edward Island, Charlottetown, PE C1A 4P3, Canada; 2 Faculty of Sustainable Design Engineering, University of Prince Edward Island, Charlottetown, PE C1A 4P3, Canada

**Keywords:** honey candies, processing, diastase, antibacterial, H_2_O_2_, anti-inflammatory

## Abstract

Raw honey naturally possesses a number of health benefits that are imbued by its components, including phenolics acids, flavonoids, enzymes, sugars, and organic acids. Processing of raw honey at high temperatures, often leads to a loss of biological activity and active ingredients, hence, reducing the medicinal benefits of processed honey samples. This study describes that the processing of raw honey into honey-based candies slightly changed the physicochemical properties of raw honey, as was observed by the loss of diastase activity and reduction in hydrogen peroxide content in honey-based candies. However, the overall health benefits of honey-based candies were maintained. The processing of honey into candies (lozenges and drops) maintained antibacterial and anti-inflammatory activities of honey candies relative to the raw honey samples.

## 1. Introduction

Honey is a supersaturated sugar solution produced naturally by honeybees from either flower nectar or honey dew. In addition to its high sugar content that makes it valuable as a sweetener, honey also contains a large variety of bioactive components including proteins, enzymes, phenolic acids, flavonoids, vitamins, minerals, and other phytochemicals [1]. These bioactive components contribute to a number of biological and medicinal capabilities including, antioxidant, antibacterial, anti-inflammatory, and wound healing activities [2].

Honey processing is routinely used at the industrial scale for the packaging and storage of honey. The processing methods vary, but standard heat treatment and filtration are typically used to improve the quality and longevity of honey; however, these processing methods also result in significant changes to the chemical and biological properties of honey [3]. Ideally the processing methods should minimize the changes to honey composition and health benefits and should prevent the loss in honey quality. The choice of honey processing method has been reported to affect colour, enzyme (diastase) activity, hydroxymethylfurfural (HMF) formation, hydrogen peroxide activity, antioxidant potential, and antibacterial power of honey samples [3].

However, commercially processed honey samples have been shown to maintain antibacterial activities in a range of bacterial species, including *S. typhi*, *P. aeruginosa*, *E. coli, S. aureus*, *B. subtilis*, *B. cereus,* and *M. luteus* [4,5]. Antibacterial activities of honey are attributed to a number of components including hydrogen peroxide formation, presence of phenolics and flavonoids, methyl glyoxal (MGO), bee defensin-1, low pH, and high osmolarity. These components in honey act synergistically to prevent bacterial growth; however, each component has a different mechanism of action and its own antibacterial strength [6]. Among the components of honey responsible for the antibacterial properties, hydrogen peroxide is often considered to be the single biggest contributor [7].

In addition to their contribution to antibacterial activities, phenolics and flavonoids are also major contributors of the anti-inflammatory activities in honey samples [2,8]. Inflammation is part of the immune response to injury or foreign agents, and may include viruses, bacteria, or chemicals. Symptoms of inflammation include redness, heat, swelling, and pain that is caused by the immune cells present at the site of infection or injury [9]. Inflammation is mediated through signalling pathways by a number of factors including nitric oxide (NO) and cytokines, such as Tumor necrosis factor-α (TNF-α), Interleukins IL-1, IL-6, etc. [2,9]. Honey has previously been shown to possess anti-inflammatory activities and specifically inhibit Nuclear Factor Kappa B (NF-κB) inflammatory pathway, which leads to the suppression of inflammatory proteins, including nitric oxide synthases (NOSs) and TNF-α [9].

Honey-based food products are gaining popularity due to the above-mentioned health properties of honey. The processing of honey into candies, gummies and jellies has previously been shown to infuse the antioxidant activities in honey-based candies [10,11]. Candies made with honey are also favourable due to their lower glycaemic index compared with sucrose, which makes them suitable for diabetics [12]. While there are extensive studies on the biological and health properties of raw and processed honey, only a handful of studies report the beneficial health properties of honey food products, including candies [10,11]. In this study, we explore the potential of honey candies (honey drops and lozenges) as antibacterial and anti-inflammatory agents. The honey derived from honey lozenges and drops was investigated for changes in diastase activity, hydrogen peroxide content, antibacterial activities, and anti-inflammatory activities. The data obtained for processed honey was then compared with raw, unprocessed honey samples.

## 2. Methods

### 2.1. Materials

The Amplex^®^ Red Hydrogen Peroxide/Peroxidase assay kit was obtained from Invitrogen, Toronto, ON, Canada, DMEM/F-12 media, and Trypsin 0.25% EDTA were obtained from Fisher Scientific, Toronto, ON, Canada. Cultures of *B. subtilis* and *E. coli* were a kind gift from Kerr Laboratory. Raw 264.7 macrophages, TNF-α, cultures of *M. luteus*, *S. epidermidis,* and *L. acidophilus* were obtained from ATCC, Toronto, ON, Canada. Nutrient broth mixture was obtained from Research Products International. HC11 mammary epithelium was obtained from ATCC, (vendor Cedarlane labs, Burlington, ON, Canada. Lipopolysaccharides (LPS) from *Escherichia coli* and penicillin–streptomycin were obtained from Millipore Sigma, Oakville, ON, Canada. MTS solution was obtained from Promega, (vendor Fisher Scientific, Toronto, ON, Canada). Research grade foetal bovine serum (FBS) was obtained from Fisher Scientific, Toronto, ON, Canada. Samples of raw honey, honey lozenges, honey drops, eucalyptus oil, and menthol were provided by Island Abbey Foods, Charlottetown, PE, Canada. The raw honey, and the honey in the lozenges and drops was multifloral honey from various producers in Canada.

### 2.2. Enzyme Activity (Diastase)

Diastase activity was determined according to the Schade method and the Harmonized methods of the International Honey commission [13]. Honey samples were prepared at a concentration of 0.2 g/mL in 0.03 M NaCl. A standardized starch solution was prepared and was calibrated to an absorbance in the desired range of 0.745–0.77, using an iodine solution. Honey and starch solutions were warmed in a 40 °C water bath before combining 5 mL of starch solution with 10 mL of honey solution. 0.5 mL aliquots of the solution were then removed every 2.5 min and were mixed with the iodine solution. The absorbance of these aliquots was determined at 660 nm using an Agilent 8453 UV–Visible spectrophotometer. A blank value was determined using deionized water in place of the starch solution.

### 2.3. Hydrogen Peroxide Analysis

Hydrogen peroxide content of honey samples was determined using the Amplex^®^ Red Hydrogen Peroxide/Peroxidase assay kit. A concentration of 0.5 g/mL of honey in 1× reaction buffer was used for the assays. The hydrogen peroxide calibration curve was prepared using concentrations of 0, 1, 2, 4, 6, 8, and 10 µM in 1× reaction buffer.

### 2.4. Antibacterial Assay

Honey samples were prepared at concentrations of 1–100 mg/mL in phosphate buffer saline (PBS). Eucalyptus oil and menthol were prepared at the concentrations found in the honey lozenge (0.24% and 0.34%, respectively) in dimethyl sulfoxide (DMSO). The final concentrations of eucalyptus oil and menthol used for this study were 2.4–240 µg/mL and 3.4–340 µg/mL, respectively. Artificial honey was prepared with 40 g of fructose and 30 g of sucrose in 18 g of water, based on the natural sugar composition of honey. Artificial honey was also prepared at concentrations of 1–100 mg/mL in PBS. Ampicillin was prepared at a concentration of 10 µM in PBS. All samples were sterilized with 0.2 μm PES syringe filters before use.

*E. coli*, *B. subtilis*, *M. luteus*, and *S. epidermidis* were cultured in nutrient broth, while *L. acidophilus* was grown in MRS broth. Bacteria were incubated at 37 °C while shaking. All bacteria were grown overnight before being diluted to an absorbance of 0.10 at 600 nm as measured with an Agilent, Mississauga, ON, Canada 8453 UV–Visible spectrophotometer and were then allowed to grow until the log phase was reached. The bacterial samples were then diluted by a factor of 10,000 before use.

The antibacterial activity of the honey samples was determined using the broth microdilution assay. The negative controls were PBS- or DMSO-treated bacterial samples. Positive controls included (1) bacterial samples treated with 10 µM ampicillin in PBS, and (2) PBS or DMSO with nutrient or De Man, Rogosa and Sharpe (MRS) broth. The raw honey, honey lozenge, honey drop, artificial honey, eucalyptus oil, and menthol samples were applied to the cultured bacterial samples. The samples were shaken and incubated at 37 °C for 24 h and the absorbance was measured at 600 nm with a Thermo Scientific Varioskan LUX, Toronto, ON, Canada microplate reader. Percent growth inhibition was determined with Equation (1):

(1) $$\%\;\mathrm{growth}\;\mathrm{inhibition}=\left(\frac{A_{\mathrm{neg}}-{\;A}_{\mathrm{sample}}}{A_{\mathrm{neg}}}\right)\times 100$$

### 2.5. Cell Culture

Raw 264.7 and HC11 cells were grown in DMEM (Dulbecco’s Modified Eagle’s Medium)/F-12 50/50 Mix. Cells were seeded in DMEM with 10% FBS and 1% penicillin–streptomycin and were incubated at 37 °C and 5% CO_2_ for 24 h before trials began. All honey samples for the nitric oxide assay (1–100 µg/mL) and TNF-α cytotoxicity assay (50–250 µg/mL) were prepared in PBS (pH 7.4) and sterilized with 0.2 μm PES syringe filters before use. Eucalyptus and menthol samples were prepared in sterile DMSO.

#### 2.5.1. Nitric Oxide Assay

The nitric oxide (NO) assay was performed based on the methods by Kassim et al. [2]. Raw 264.7 cells were seeded into 96-well plates at a density of 100,000 cells/well. Cells were incubated at 37 °C and 5% CO_2_ overnight before being treated with honey samples between the concentrations of 1–100 µg/mL in PBS, and with the controls of PBS, eucalyptus oil (2.4–240 ng/mL) and menthol (3.4–340 ng/mL). Cells were treated with honey samples for 24 h before stimulation with 1 µg/mL LPS. Untreated cells were used as negative controls. 24 h after LPS stimulation, media was removed from the plates and NO content was determined with the Griess assay. Briefly, 150 µL of cell culture media was mixed with 130 µL of deionized water and 20 µL of Griess reagent and was left for 30 min at room temperature. Absorbance of the solution was measured at 548 nm with a Thermo Scientific Varioskan LUX microplate reader and relative nitric oxide concentration was determined with a standard curve of sodium nitrite (0–100 µM).

#### 2.5.2. Cell Viability of Raw 264.7

Cell viability of Raw 264.7 cells was determined under the conditions of the nitric oxide assay. Following 48 h of incubation and treatment, cell viability was determined by MTS assay. Media was removed, then cells were washed with PBS, and media was replaced with media containing 4% MTS solution. The cells were then incubated for 3 h before the absorbance was measured at 490 nm with a Thermo Scientific Varioskan LUX microplate reader. Cell viability was determined with Equation (1).

#### 2.5.3. TNF-α Cytotoxicity Assay

The TNF-α cytotoxicity assay was performed based on the methods by Kassim et al. [2]. HC-11 fibroblasts were seeded into 96-wells plates at the density of 50,000 cells/well. Cells were incubated overnight at 37 °C and 5% CO_2_ after which they were treated with honey at concentrations ranging from 50–250 µg/mL in PBS, as well as with controls including PBS, eucalyptus oil (120–600 ng/mL), and menthol (170–850 ng/mL). 24 h after the treatment with honey samples, the cells were treated with TNF-α at a final concentration of 1 ng/mL. Untreated cells were used as a negative control. The cells were incubated for 24 h, the media was then removed, and MTS assay was performed to determine the cell viability. The MTS assay was performed by replacing media with 100 µL of 4% MTS solution in media. The cells were then incubated for 3 h before the absorbance was measured at 490 nm with a Thermo Scientific Varioskan LUX microplate reader.

### 2.6. Statistical Analysis

All the assays were carried out in triplicate and values were expressed as means ± standard deviation. For statistical analysis, a one-way ANOVA with post hoc t-tests and a Bonferroni correction was performed using Microsoft Excel. A *p* value of less than 0.05 was considered significant.

## 3. Results and Discussion

Honey possesses a number of beneficial health properties, including antioxidant, antibacterial, and anti-inflammatory activities. These health properties originate from the unique chemical composition of honey, namely, sugars, enzymes, phenolics, and flavonoids, which contribute towards the biological properties of honey. Enzymes such as diastase may come from nectar or are incorporated by bees during the production of honey but can also be diminished or destroyed during commercial processing. Similarly, other enzymes, glucose oxidase and catalase, are responsible for the production and destruction of hydrogen peroxide in honey and contribute towards antibacterial properties. Phenolics and flavonoids contribute to many of the health benefits of honey, including the antioxidant, antibacterial (non-peroxide activity), and anti-inflammatory properties. The synergistic action of different components of honey towards the antibacterial and anti-inflammatory activities ensure superior health properties of this natural liquid; however, processing of honey at high temperatures, under pressure, or during filtration can compromise the health benefits of the resultant honey-based products [1,2,4,7,9,14,15,16].

### 3.1. Enzymatic Activity of Honey Lozenges and Drops

Enzymatic activity of fresh honey is often measured in the form of diastase activity (termed as the diastase number or DN) and is dependent upon the type and origin of the honey samples. Diastase is an enzyme that catalyses the breakdown of starch into maltose. The diastase activity of multifloral raw honey, the source of honey to make honey lozenges and drops, was measured to be 29.1 ± 0.2 DN (Supplementary Figure S1), well within the range of reported values for raw honey. For example, Pasias et al. showed that the diastase activity of 39 blossom and honeydew samples obtained from Greece ranged from 2.4–51 DN [17].

Processing of honey samples by excessive heating is well documented to reduce the diastase activity of honey samples. For example, Tosi et al. found that processing of honey at 100 °C resulted in complete loss of diastase activity in six multifloral honeys obtained from Argentina [18]. As expected, processing of raw honey into lozenges and drops resulted in complete loss of diastase activity (Supplementary Figure S1), as they are produced by vacuum drying at a temperature of 98 °C and at a pressure of at least 27 inHg [19].

Irreversible loss of diastase activity in the honey lozenge and drop indicates that heat treatment may have also resulted in the loss of enzymatic activity of other key enzymes present in the honey, such as glucose oxidase or catalase. Glucose oxidase converts glucose into hydrogen peroxide and gluconic acid. Hydrogen peroxide is naturally present in honey in small quantities and is believed to protect unfinished honey from spoilage during its production by bees. The denaturation temperature for free glucose oxidase is 55.8 ± 1.2 °C, suggesting its potential denaturation at high temperatures (98 °C is required for honey drop and lozenge formulation) [19,20]. Deactivation of glucose oxidase during honey lozenge and drop formulation is expected to decrease the hydrogen peroxide concentration in processed honey samples, as compared with the raw honey.

**Figure 1:**
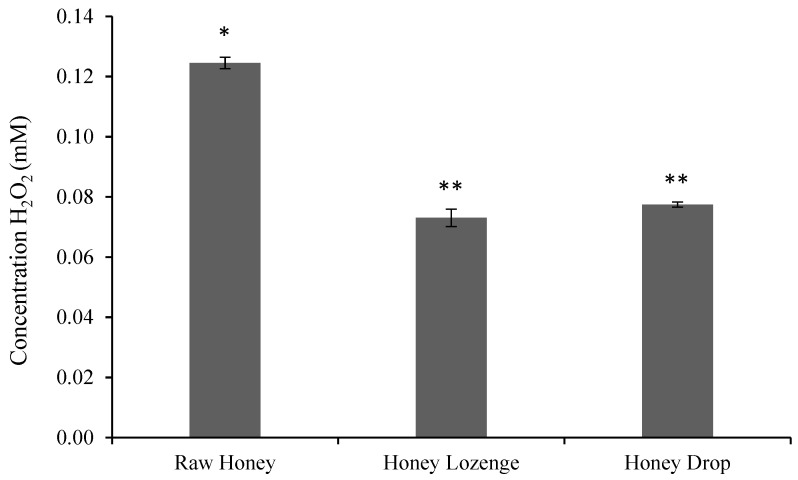
Hydrogen peroxide concentration in honey samples (mM/g of honey), as determined by Amplex^®^ Red Hydrogen Peroxide/Peroxidase assay. The assay was performed at a concentration of 0.5 g honey/mL of reaction buffer. Bars with a different number of symbols (* and ** represent statistically different samples).

In this study, hydrogen peroxide content of raw honey was found to be 0.125 ± 0.002 mM honey for a 50% weight/volume *(w/v)* honey solution (Figure 1) and is in agreement with the values reported by others. For example, hydrogen peroxide values for buckwheat honey were found to be between 0.25 ± 0.02 and 2.68 ± 0.04 mM for a 25% *w/v* sample by Brudzynski et al. [7]. Similarly, Chen et al. also showed that hydrogen peroxide values of spotted gum and red stringybark honeys ranged from 0.15 ± 0.001 to 1.017 ± 0.01 mM for a 50% *w/v* sample [21].

The formulation of honey into candies reduced the overall hydrogen peroxide content and was found to be 0.073 ± 0.003 mM and 0.077 ± 0.001 mM of honey in lozenges and drop, respectively, with 50% *w/v* samples (Figure 1). Chen et al. also showed that the hydrogen peroxide content of Australian origin honey samples significantly reduced after filtration and processing at 45 °C for 8 h. The processing of honey reduced the hydrogen peroxide content in 10 out of 11 samples, and at least four of these samples showed complete loss of hydrogen peroxide content [21].

### 3.2. Antibacterial Activities of Honey Lozenges and Drops

Hydrogen peroxide is considered to be one of the greatest contributors to the antibacterial activities of honey; however, data suggests that levels of H_2_O_2_ and antimicrobial action of honey samples do not always correlate [21]. Hence, further evaluation of antimicrobial potentials of honey-based candies was necessary, despite the lower concentration of H_2_O_2_ in the honey lozenge and drop compared to raw honey. Honey contains several other active ingredients, including phenolics and flavonoids, methyl glyoxal (MGO), bee defensin-1, low pH, and high osmolarity that also contribute towards its antimicrobial activity [6].

**Figure 2:**
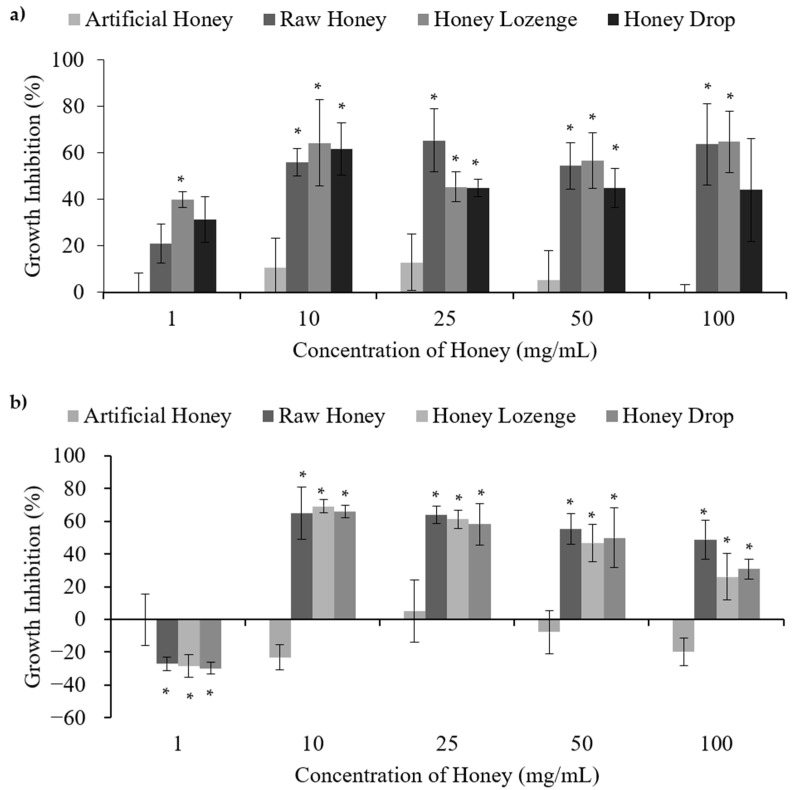
Antibacterial activities of honey with (**a**) *E. coli* and (**b**) *M. luteus*, upon treatment with different concentrations of raw and processed honey samples. Artificial honey is included as a control for the sugar content of honey. * Indicates significant difference from the negative control.

**Figure 3:**
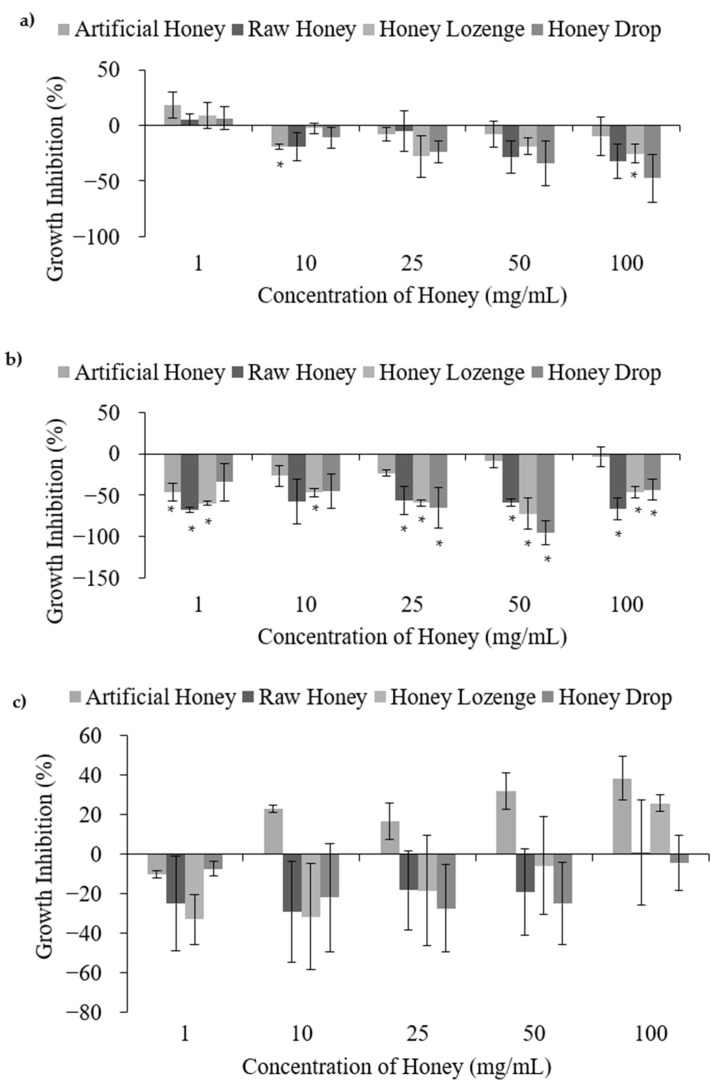
Pro-bacterial activities of honey with (**a**) *B. subtilis*, (**b**) *S. epidermidis*, and (**c**) *L. acidophilus*, upon treatment with different concentrations of raw and processed honey samples. Artificial honey is included as a control for the sugar content of honey. * Indicates significant difference from the negative control.

Antibacterial action of raw honey, honey lozenges, and honey drops was studied in Gram-negative bacterium—*Escherichia coli*, Gram-variable bacterium—*Micrococcus luteus,* and Gram-positive bacteria—*Bacillus subtilis*, *Staphylococcus epidermidis*, and *Lactobacillus acidophilus.* (Figure 2 and Figure 3, Supplementary Table S1). These bacteria were chosen as examples of harmful or infectious bacteria as well as beneficial or probiotic bacteria. Artificial honey was used as a control to simulate the sugar content of honey that may cause osmotic stress and thereby contribute to bacterial death. Eucalyptus oil and menthol at the concentrations found in the lozenge (0.24% and 0.34% mass, respectively) were also included as controls. Overall, our results indicate that processing of honey into honey lozenges and drops had no significant effect on the antibacterial activities and that the raw honey, honey lozenge, and honey drop showed bacterial species dependent antibacterial activities.

*E. coli* is a bacterium that occurs naturally in the lower-intestine but may also result in infection causing severe diarrhoea such is caused by the O157:H7 strain. Raw honey, honey lozenge, and honey drop showed concentration dependent bacterial death in the case of *E. coli.* As shown in Figure 2a, treatment of bacteria at 1 mg/mL resulted in no cell death. However, statistically significant antibacterial activities were observed relative to the negative control upon the treatment of *E. coli* with 10–100 mg/mL of the raw honey, honey lozenge, and honey drop. The maximum inhibition of bacterial growth for each of the honey samples was 65.3%, 64.7%, and 61.4% for the raw honey, honey lozenge, and honey drop, respectively, while the artificial honey had a maximum inhibition of only 12.7%. Menthol and eucalyptus oil showed 13.7%, and 14.8% bacterial inhibition, at the concentrations present in the honey lozenge, indicating that these medicinal ingredients could have partially contributed to the antibacterial activities of the honey lozenge (Supplementary Figure S2a). MIC values obtained for raw honey, honey lozenge and honey drop are in agreement with the previously reported literature. For example, Zainol et al. investigated the effect of Acacia, Gelam, Kelulut, Pineapple, Tualang, and Manuka honey on *E. coli* cell viability and determined that the MIC_50_ for these samples ranged from 1.6–6.3% *w/v*. Minimal Inhibitory Concentration (MIC_99_) for these honey samples were found to be between 12.5–25% *w/v* [22]. Chauhan et al. reported MIC values of 0.625–1.25 mg/mL for methanol extracts and 2.5 mg/mL for ethanol extracts of raw and processed honey samples from India, respectively [4].

Most strains of *M. luteus* are harmless, but it is considered an opportunistic pathogen, which can cause serious infections. Similar to the results for *E. coli*, treatment of *M. luteus* with the honey samples at concentrations between 10–100 mg/mL resulted in significant antibacterial activities relative to the negative control (Figure 2b). The maximum bacterial growth inhibition of each of the honey samples was 65.0%, 69.2%, and 66.0% for the raw honey, honey lozenge, and honey drop, respectively, while the artificial honey had a maximum growth inhibition of only 5.2%. MIC of 0.625 mg/mL for *M. luteus* was reported with methanolic extracts of raw and processed honey in the literature [5]. Treatment of *M. luteus* with the eucalyptus oil and menthol resulted in maximum growth inhibition of 11.8% and 20.9%, which were not significantly different from the negative control (Supplementary Figure S2b). MIC of *M. longifolia*, which are largely composed of menthol, against *M. luteus* is reported to be 0.19 mg/mL [23].

Treatment of *B. subtilis, S. epidermidis and L. acidophilus* at all studied concentrations of raw honey, honey lozenge and drop showed no antibacterial activities (Figure 3). *B. subtilis* is a benign bacterium commonly used as a model for Gram-positive bacteria; however, it may cause infection in immunocompromised individuals. A positive growth effect was observed for *B. subtilis* treated with honey samples; however, increased bacterial growth was not significant apart from the honey lozenge at 100 mg/mL (Figure 3a). The increase in *B. subtilis* growth in the presence of honey samples is well-documented by others. Brudzynski et al. reported increased growth of *B. subtilis* at honey concentrations ranging from 1.56% to 12.5% volume/volume *(v/v)* [7]. Similarly, Zainol et al. reported increased growth of *B. cereus* for acacia and pineapple honeys at concentrations less than 5% *w/v* and 2% *w/v*, respectively [22]. Treatment of *B. subtilis* with the eucalyptus and menthol samples did not exhibit significant antibacterial activities at any of the studied concentrations (Supplementary Figure S2c).

*S. epidermidis* is typically benign and has low pathogenicity, but it is a common cause of infection of indwelling medical devices [24]. *S. epidermidis* has also been documented for its probiotic activities by preventing the colonization of other bacteria, including *S. aureus* and *P. acnes* [24,25]. Treatment of *S. epidermidis* with the processed and raw honey samples showed an overall increase in bacterial growth relative to the negative control at most of the studied concentrations (Figure 3b). The antibacterial efficacies of honey against *S. epidermidis* are reported to be variable and may depend on the type and the origin of the honey samples [26,27]. Basualdo et al. showed that 6 out of 10 honey samples possessed antibacterial activities against *S. epidermidis*, but only when the honey was undiluted [27]. Similarly, Baltrusaityte et al. reported that honey required a concentration of at least 25% in order to achieve reasonable antibacterial activity against *S. epidermidis* [26]. In this study lower concentrations (up to 10% *w/v*) were used due to the high viscosities of honey samples extracted from the honey drop and lozenge, preventing filter sterilization at higher concentrations. Eucalyptus oil and menthol demonstrated significant antibacterial activities against *S. epidermidis* relative to the negative control at the two highest concentrations investigated (Supplementary Figure S2d). These results are comparable to the MIC values reported elsewhere. For example, Desam et al. reported an MIC of 23.0 ± 0.02 µg/mL for menthol [28], and Göger et al. reported an MIC of 625 µg/mL for *Eucalyptus globulus* essential oil with *S. epidermidis* [29]. Despite the strong antibacterial action of eucalyptus and menthol against *S. epidermidis*, lower concentrations of these extracts in honey lozenge did not contribute towards antibacterial activities, indicating strong pro-bacterial activity of this honey-based candy.

*L. acidophilus* is a very commonly used probiotic bacterium. It showed an overall positive growth affect in the presence of the honey samples (Figure 3c); however, large standard deviations also indicate possible indifference from the negative control. Unfortunately, abnormal distribution of the data for *L. acidophilus* prevented the use of ANOVA and t-tests to confirm the significance from the negative control. Shamala et al. reported a comparable growth rate of *L. acidophilus* in both honey containing media and media containing other sugars (glucose and lactose) [30], which is in agreement with our results showing comparable growth of *L. acidophilus* in all the samples, including honey, artificial honey and untreated media. The results showed that eucalyptus oil exhibited no significant bacterial death compared to the negative control; however, menthol demonstrated significant antibacterial activities with a maximum percent inhibition of 77.0% at 340 µg/mL (Supplementary Figure S2e). Antibacterial activity of menthol against *L. acidophilus* has also been reported in the literature, and Al-Bayati determined an MIC of 31.2 µg/mL for menthol isolated from *M. longifolia* leaf oil [31]. The antibacterial action of menthol against *L. acidophilus* may have contributed to the decreased bacterial viability at 100 mg/mL of the honey lozenge, as compared with the other honey samples.

Overall, the processed honey candies maintained antibacterial and pro-bacterial activities relative to the raw, unprocessed honey. This is in agreement with the literature, where processed honey samples have been shown to maintain antibacterial activities against a number of bacteria, including *S. typhi*, *P. aeruginosa*, *E. coli, S. aureus*, *B. subtilis*, *B. cereus,* and *M. luteus* [4,5]. In our study, a lower hydrogen peroxide content of the honey candies did not significantly reduce the antibacterial activities compared to the raw honey, possibly due to the contribution of non-peroxide factors, including phenolics.

### 3.3. Anti-Inflammatory Activities of Honey Drops and Lozenges

Anti-inflammatory agents block the pro-inflammatory pathways in the body and act to reduce inflammation, including redness, swelling, and pain. Honey is an anti-inflammatory agent that downregulates inflammatory transcription factors, suppresses the production of pro-inflammatory cytokines, and stimulates the production of inflammatory mediators [16]. Typical in vitro methods for the determination of anti-inflammatory activities of honey include the investigation of the effect of honey on nitric oxide levels and the production of pro-inflammatory cytokines in the presence of allergens, such as lipopolysaccharides (LPS) [2,32,33,34]. In this study, anti-inflammatory activities of the raw honey, honey lozenge, and honey drop were determined by the inhibition of nitric oxide production in Raw 264.7 cells and by measuring the prevention of TNF-α cytotoxicity in HC11 cells.

#### 3.3.1. Nitric Oxide Inhibition Assay

Nitric oxide is a signalling molecule that is involved in the progression of inflammation and is produced by inducible nitric oxide synthase (iNOS) enzyme. Honey is known to inhibit nitric oxide production and exhibit anti-inflammatory activities by the suppression of the nuclear factor-κB (NF-κB) pathway, hence inhibiting inflammatory factors, such as interleukin 6 (IL-6), tumor necrosis factor (TNF), cyclooxygenase-2 (COX-2), and inducible nitric oxide synthase (iNOS) [9]. Anti-inflammatory activities and inhibition of the NF-κB pathway by honey is believed to be due to the action of phenolics and flavonoids. For example, quercetin and kaempferol have been found to decrease iNOS, COX-2, and CRP protein concentrations, and also decrease NF-κB activation and phosphorylated IκB-α and IKK concentration in CHL (Chang liver) cells [8]. We have previously shown that honey lozenges and drops possess a significantly higher phenolic content than raw honey [10]. Given the role of honey phenolics in anti-inflammatory activities, the significantly higher phenolic content of the honey lozenge and drop, warrants their evaluation for anti-inflammatory activities.

**Figure 4:**
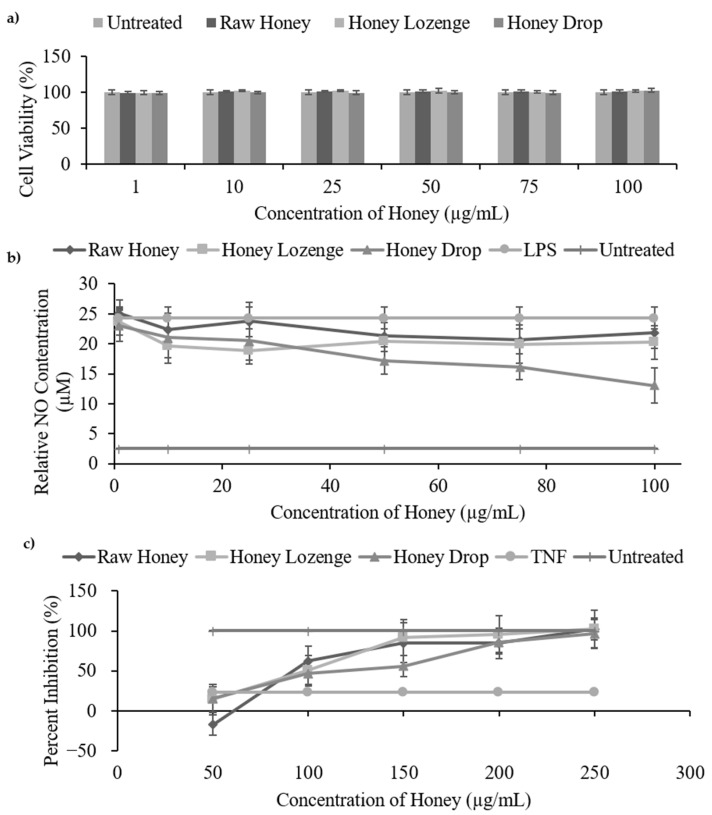
In vitro anti-inflammatory assays of honey samples. (**a**) Cell viability of Raw 264.7 macrophages treated with honey samples (1–100 µg/mL in PBS). (**b**) Nitric oxide inhibition assay measures the ability of the honey samples (1–100 µg/mL in PBS) to inhibit the production of nitric oxide by Raw 264.7 macrophages that were stimulated by LPS (1 µg/mL). (**c**) TNF-α cytotoxicity assay.

A nitric oxide inhibition assay was used to study the anti-inflammatory activities of the honey lozenge and drop and was compared with the raw honey samples. In general, our results show that macrophages stimulated with lipopolysaccharide produced less nitric oxide in the presence of the raw honey, honey lozenge, and honey drop (Figure 4b, Supplementary Table S2).

The minimum nitric oxide production for the raw honey, honey lozenge, and honey drop were 20.70 µM, 19.96 µM, and 13.02 µM, respectively, compared with the LPS sample which produced 24.30 µM of NO and the untreated sample which produced only 2.55 µM of NO. These values represent a maximum percent inhibition of 14.82% for raw honey, 22.29% for the honey lozenge, and 46.40% for the honey drop. Kassim et al. reported a maximum nitric oxide inhibition of ~16 µM (~80%) for honey ethyl acetate extracts and ~5.5 µM (~40%) for honey methanol extracts at concentrations ranging from 3.125–100 µg/mL [2]. Biluca et al. reported percent nitric oxide inhibition from 25.8% to 37.7% for stingless bee honey from Brazil at concentrations of 10–100 µg/mL [32].

In this study, there was no significant difference in the nitric oxide inhibition between the honey samples, with the exception of the honey drop (46.6%) showing significantly higher inhibition (*p* < 0.01) than the raw honey (10.31%) at 100 µg/mL. Due to the well-documented role of phenolics and flavonoids in anti-inflammatory activities, the greater inhibition of nitric oxide production by the honey drop compared with the raw honey may be attributed to its greater total phenolic content [8,10]. Eucalyptus oil and menthol controls, at the concentrations present in the honey lozenge, had no significant effect on the production of nitric oxide (Supplementary Figure S3b).

A cell viability assay of Raw 264.7 macrophages was conducted to ensure that the observed reduction in nitric oxide production was due to anti-inflammatory activities and not due to the unwanted cell death upon treatment with honey samples. As shown in Figure 4a, Raw 264.7 cells maintained their cell viability (90–100%) at all of the studied concentrations (1–100 µg/mL) of the raw honey, honey drop and honey lozenge and there was no significant cell death 24 h post-treatment (Figure 4a). Eucalyptus oil and menthol controls also exhibited no changes in cell viability at the studied concentrations (Supplementary Figure S3a). Overall, this indicates that the observed reduction in nitric oxide production was due to the inhibition of inflammatory pathways rather than the toxicity of samples causing cell death. Our results are also in agreement with other studies showing that honey is not toxic to Raw 264.7 macrophages at concentrations below 100 µg/mL [2,32].

#### 3.3.2. Inhibition of TNF-α Cytotoxicity

TNF-α is a pro-inflammatory cytokine that is produced by macrophages and is capable of inducing cytotoxicity by reactive oxygen species (ROS) production and by free radical formation, causing cell damage and death. Honey is well documented to prevent TNF-α cytotoxicity through its radical scavenging and antioxidant properties [2,35,36]. Phenolics and flavonoids are major contributors to antioxidant and free radical scavenging capabilities of honey and antioxidant strength of phenolics is related to the number of hydroxyl groups present in the molecule [15]. TNF-α cytotoxicity inhibition effects of honey have previously been linked with phenolic (protocatechuic acid and caffeic acid) and flavonoid (kaempferol and quercetin) content [37]. Given the high concentrations of phenolics and flavonoids in raw honey, honey lozenges, and honey drops [10], the role of raw honey and honey-based candies in preventing TNF-α cytotoxicity, was evaluated.

Our results indicate that the raw honey, honey lozenge, and honey drop were capable of inhibiting cytotoxicity in HC11 cells caused by TNF-α, as was measured by the MTS cell viability assay (Figure 4c, Supplementary Table S3). The raw honey, honey lozenge, and honey drop significantly inhibited cell cytotoxicity, at the concentrations ranging from 150–250 µg/mL in HC11 cells, relative to the TNF-α control (*p* < 0.02). Half maximal effective concentration (EC_50_) values for TNF-α cytotoxicity inhibition for the raw honey, honey lozenge, and honey drop were determined to be 156.1, 136.2, and 154.7 µg/mL, respectively. These values are comparable with EC_50_ values reported by Kassim et al., who reported EC_50_ of 168.1 µg/mL for methanol extracts of honey and 235.4 µg/mL for ethyl acetate extracts of honey [2]. The eucalyptus oil and menthol controls did not demonstrate significant inhibition of HC11 cytotoxicity (Supplementary Figure S3c) [10], further highlighting the key role of phenolics and flavonoids on anti-inflammatory activities of honey samples.

## 4. Conclusions and Future Directions

In conclusion, while a loss of diastase activity and decrease in H_2_O_2_ content during the processing of honey into drops and lozenges may indicate a potential loss of quality and health benefits, honey candies overall maintained their health properties. The honey lozenge and drop were found to maintain antibacterial activities against *E. coli* and *M. luteus*, and exhibited pro-bacterial activities against *B. subtilis*, *S epidermidis*, and *L. acidophilus.* The antibacterial and pro-bacterial results obtained for honey candies were comparable with that of the raw honey source. The honey candies also maintained or enhanced the anti-inflammatory activities of raw honey as was measured by nitric oxide inhibition and TNF-α cytotoxicity inhibition assays. The improved anti-inflammatory activities of the honey candies were attributed to the higher phenolic content and antioxidant activities of honey candies, when compared with raw honey. Hence, these candies may offer intrinsic and enhanced health properties of honey. Further investigation of the biological and health properties of honey candies will include antifungal, antiviral, and wound healing properties of honey candies, relative to raw honey.

## Data Availability

Not applicable.

## Supplementary Materials

The following supporting information can be downloaded at: https://www.mdpi.com/article/10.3390/nu14224738/s1, Figure S1: Diastase activity (DN) of honey samples. (*) Indicates complete absence of diastase activity; Figure S2: Bacterial activity of eucalyptus oil (2.4−240 µg/mL) and menthol (3.4−340 µg/ml) with (a) *E. coli*, (b) *M. luteus*, (c) *B. subtilis*, (d) *S. epidermidis*, and (e) *L. acidophilus*; Figure S3: In vitro anti−inflammatory assays of eucalyptus oil and menthol controls. (a) Cell viability of Raw 264.7 macrophages treated with eucalyptus and menthol (0.24% and 0.34% of honey concentration respectively). (b) Nitric oxide inhibition assay; Table S1: Table of Values—Activity of honey samples and controls with *E. coli*, *B. subtilis*, *M. luteus*, *S. epidermidis*, and *L. acidophilus*; Table S2: Nitric oxide production by Raw 264.7 cells (µM) when exposed to honey samples (1–100 µg/mL) and controls; Table S3: Percent inhibition of TNF−α cytotoxicity in HC11 cells by honey samples (50–250 µg/mL) and controls.

## References

[1] Alvarez-Suarez J.M., Gasparrini M., Forbes-Hernández T.Y., Mazzoni L., Giampieri F. (2014). The Composition and Biological Activity of Honey: A Focus on Manuka Honey. Foods.

[2] Kassim M., Achoui M., Mustafa M.R., Mohd M.A., Yusoff K.M. (2010). Ellagic acid, phenolic acids, and flavonoids in Malaysian honey extracts demonstrate in vitro anti-inflammatory activity. Nutr. Res..

[3] Subramanian R., Hebbar H.U., Rastogi N. (2007). Processing of Honey: A Review. Int. J. Food Prop..

[4] Chauhan A., Pandey V., Chacko K.M., Khandal R.K. (2010). Antibacterial activity of raw and processed honey. Electron. J. Biol..

[5] Mohapatra D.P., Thakur V., Brar S.K. (2010). Antibacterial Efficacy of Raw and Processed Honey. Biotechnol. Res. Int..

[6] Kwakman P.H.S., te Velde A.A., de Boer L., Speijer D., Vandenbroucke-Grauls M.J.C., Zaat S.A.J. (2010). How honey kills bacteria. FASEB J..

[7] Brudzynski K., Abubaker K., Laurent M., Castle A. (2011). Re-Examining the Role of Hydrogen Peroxide in Bacteriostatic and Bactericidal Activities of Honey. Front. Microbiol..

[8] García-Mediavilla M.V., Crespo I., Collado P.S., Esteller A., Sánchez-Campos S., Tuñón M.J., González-Gallego J. (2007). The anti-inflammatory flavones quercetin and kaempferol cause inhibition of inducible nitric oxide synthase, cyclooxygenase-2 and reactive C-protein, and down-regulation of the nuclear factor kappaB pathway in Chang Liver cells. Eur. J. Pharmacol..

[9] Hussein S.Z., Yusoff K.M., Makpol S., Yusof Y.A.M. (2013). Gelam Honey Attenuates Carrageenan-Induced Rat Paw Inflammation via NF-κB Pathway. PLoS ONE.

[10] Larsen P., Ahmed M. (2022). Evaluation of antioxidant potential of honey drops and honey lozenges. Food Chem. Adv..

[11] Rivero R., Archaina D., Sosa N., Leiva G., Coronel B.B., Schebor C. (2020). Development of healthy gummy jellies containing honey and propolis. J. Sci. Food Agric..

[12] Chepulis L., Starkey N. (2008). The Long-Term Effects of Feeding Honey Compared with Sucrose and a Sugar-Free Diet on Weight Gain, Lipid Profiles, and DEXA Measurements in Rats. J. Food Sci..

[13] Bogdanov S. (2002). Harmonised Methods of the International Honey Commission.

[14] Ball D.W. (2007). The Chemical Composition of Honey. J. Chem. Educ..

[15] Cianciosi D., Forbes-Hernández T.Y., Afrin S., Gasparrini M., Reboredo-Rodriguez P., Manna P.P., Zhang J., Bravo Lamas L., Martínez Flórez S., Agudo Toyos P. (2018). Phenolic Compounds in Honey and Their Associated Health Benefits: A Review. Molecules.

[16] Ranneh Y., Akim A.M., Hamid H.A., Khazaai H., Fadel A., Zakaria Z.A., Albujja M., Abu Bakar M.F. (2021). Honey and its nutritional and anti-inflammatory value. BMC Complement. Med. Ther..

[17] Pasias I.N., Kiriakou I.K., Proestos C. (2017). HMF and diastase activity in honeys: A fully validated approach and a chemometric analysis for identification of honey freshness and adulteration. Food Chem..

[18] Tosi E., Martinet R., Ortega M., Lucero H., Ré E. (2008). Honey diastase activity modified by heating. Food Chem..

[19] Schade J.E., Marsh G.L., Eckert J.E. (1958). Diastase activity and hydroxy-methyl-furfural in honey and their usefulness in detecting heat alteration. J. Food Sci..

[20] Zoldak G., Zubrik A., Musatov A., Stupák M., Sedlak E. (2004). Irreversible Thermal Denaturation of Glucose Oxidase from Aspergillus niger Is the Transition to the Denatured State with Residual Structure. J. Biol. Chem..

[21] Chen C., Campbell L.T., Blair S.E., Carter D.A. (2012). The effect of standard heat and filtration processing procedures on antimicrobial activity and hydrogen peroxide levels in honey. Front. Microbiol..

[22] Zainol M.I., Yusoff K.M., Yusof M.Y.M. (2013). Antibacterial activity of selected Malaysian honey. BMC Complement. Altern. Med..

[23] Hafedh H., Fethi B.A., Mejdi S., Emire N., Amina B. (2010). Effect of mentha longifolia L. ssp longifolia essential oil on the morphology of four pathogenic bacteria visualized by atomic force microscopy. Afr. J. Microbiol. Res..

[24] Otto M. (2009). Staphylococcus epidermidis—The ‘accidental’ pathogen. Nat. Rev. Genet..

[25] Wang Y., Kuo S., Shu M., Yu J., Huang S., Dai A., Two A., Gallo R.L., Huang C.-M. (2013). *Staphylococcus epidermidis* in the human skin microbiome mediates fermentation to inhibit the growth of *Propionibacterium acnes*: Implications of probiotics in acne vulgaris. Appl. Microbiol. Biotechnol..

[26] Baltrusaityte V., Venskutonis P.R., Ceksteryte V. (2007). Antibacterial activity of honey and beebread of different origin against S. aureus and S. epidermidis. Food Technol. Biotechnol..

[27] Basualdo C., Sgroy V., Finola M.S., Marioli J.M. (2007). Comparison of the antibacterial activity of honey from different provenance against bacteria usually isolated from skin wounds. Veter. Microbiol..

[28] Desam N.R., Al-Rajab A.J., Sharma M., Mylabathula M.M., Gowkanapalli R.R., Albratty M. (2019). Chemical constituents, in vitro antibacterial and antifungal activity of *Mentha* × *Piperita* L. (peppermint) essential oils. J. King Saud. Univ. Sci..

[29] Göger G., Karaca N., Altınbaşak B.B., Demirci F. (2020). In vitro antimicrobial, antioxidant and anti-inflammatory evaluation of eucalyptus globulus essential oil. Nat. Volatiles Essent. Oils.

[30] Shamala T.R., Shri Jyothi Y., Saibaba P. (2000). Stimulatory effect of honey on multiplication of lactic acid bacteria under in vitro and in vivo conditions. Lett. Appl. Microbiol..

[31] Al-Bayati F.A. (2009). Isolation and identification of antimicrobial compound from Mentha longifolia L. leaves grown wild in Iraq. Ann. Clin. Microbiol. Antimicrob..

[32] Biluca F.C., da Silva B., Caon T., Mohr E.T.B., Vieira G.N., Gonzaga L.V., Vitali L., Micke G., Fett R., Dalmarco E.M. (2020). Investigation of phenolic compounds, antioxidant and anti-inflammatory activities in stingless bee honey (Meliponinae). Food Res. Int..

[33] Silva B., Biluca F.C., Gonzaga L.V., Fett R., Dalmarco E.M., Caon T., Costa A.C.O. (2021). In vitro anti-inflammatory properties of honey flavonoids: A review. Food Res. Int..

[34] Sun L.-P., Shi F.-F., Zhang W.-W., Zhang Z.-H., Wang K. (2020). Antioxidant and Anti-Inflammatory Activities of Safflower (*Carthamus tinctorius* L.) Honey Extract. Foods.

[35] Goossens V., Grooten J., De Vos K., Fiers W. (1995). Direct evidence for tumor necrosis factor-induced mitochondrial reactive oxygen intermediates and their involvement in cytotoxicity. Proc. Natl. Acad. Sci USA.

[36] Larrick J.W., Wright S.C. (1990). Cytotoxic mechanism of tumor necrosis factor-α. FASEB J..

[37] Habtemariam S. (2000). Natural Inhibitors of Tumour Necrosis Factor-α Production, Secretion and Function. Planta Med..

