# Supplementary material for: Evaluation of Biological Activities and Medicinal Properties of Honey Drops and Honey Lozenges
Source: Nutrients. 2022 Nov 10;14(22):4738. doi: 10.3390/nu14224738 (PMC9693101; doi:10.3390/nu14224738)
Supplement: Supplementary file 1 [file nutrients-14-04738-s001.zip › nutrients-2022289-supplementary.pdf]

nutrients

Evaluation of Biological Activities and Medicinal Properties of Honey Drops and Honey Lozenges

Petra Larsen[1] and Marya Ahmed[1,2,*]

Supplementary Materials

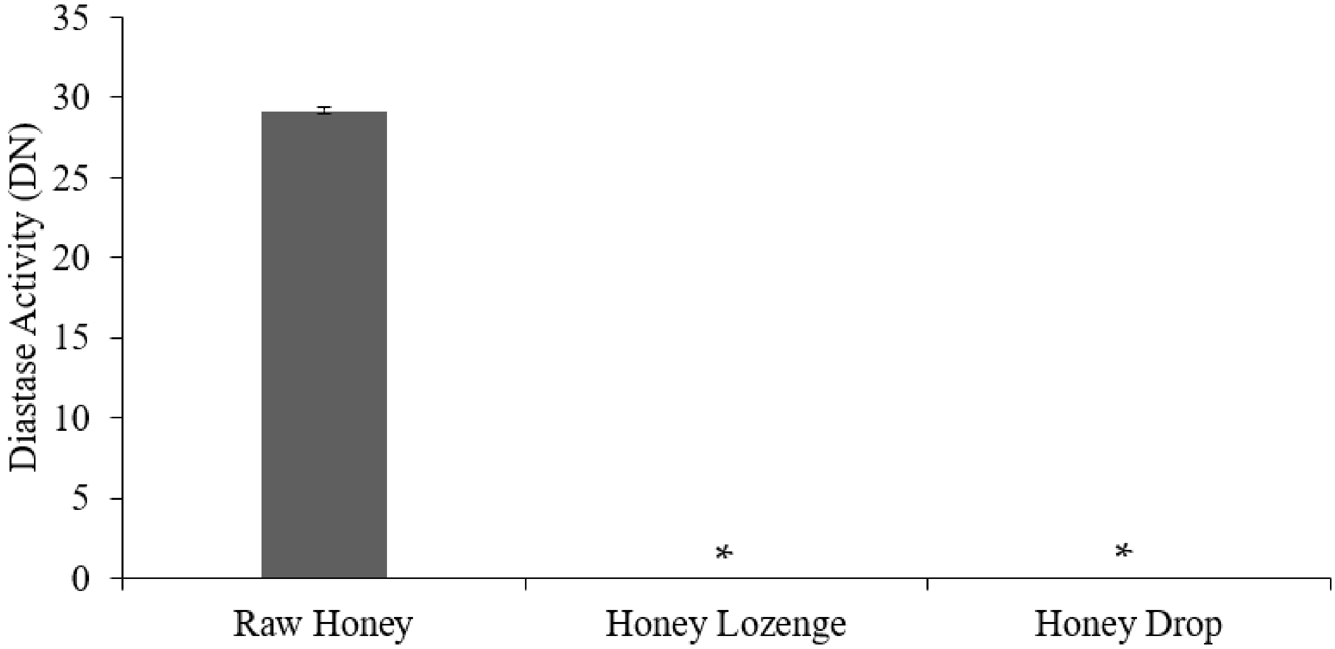

Supplementary Figure S1. Diastase activity (DN) of honey samples. (*) Indicates complete absence of diastase activity.

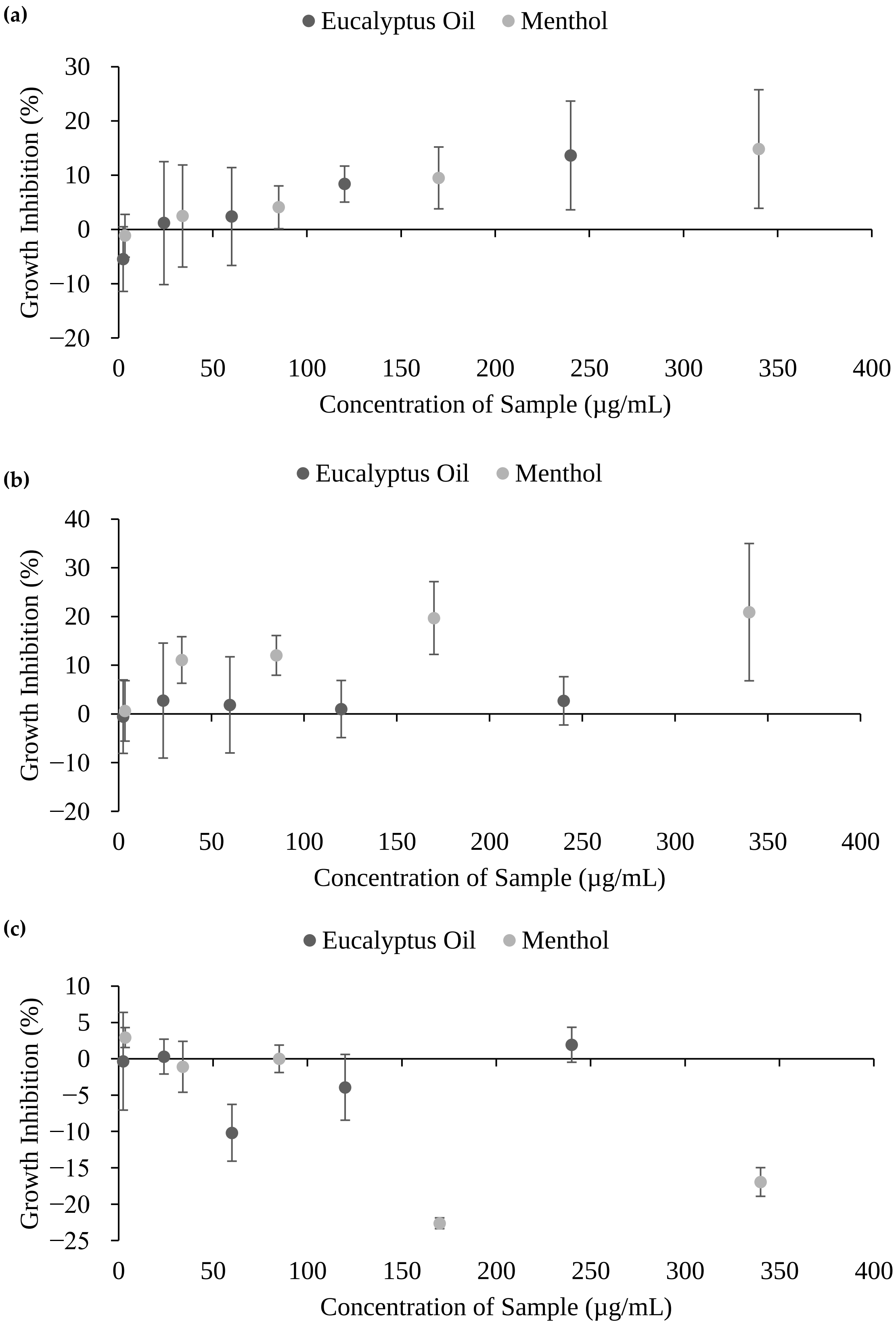
(a)
Eucalyptus Oil
Menthol
Growth Inhibition (%)
30
20
10
0
−10
−20
0
50
100
150
200
250
300
350
400
Concentration of Sample (μg/mL)
(b)
Eucalyptus Oil
Menthol
Growth Inhibition (%)
40
30
20
10
0
−10
−20
0
50
100
150
200
250
300
350
400
Concentration of Sample (μg/mL)
(c)
Eucalyptus Oil
Menthol
Growth Inhibition (%)
10
5
0
−5
−10
−15
−20
−25
0
50
100
150
200
250
300
350
400
Concentration of Sample (μg/mL)

(d)

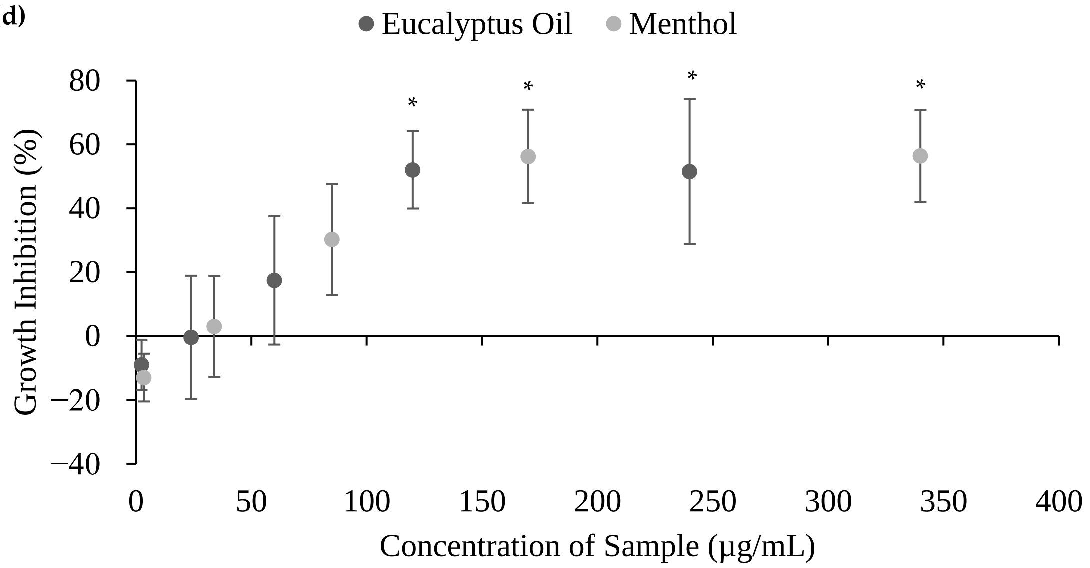

(e)

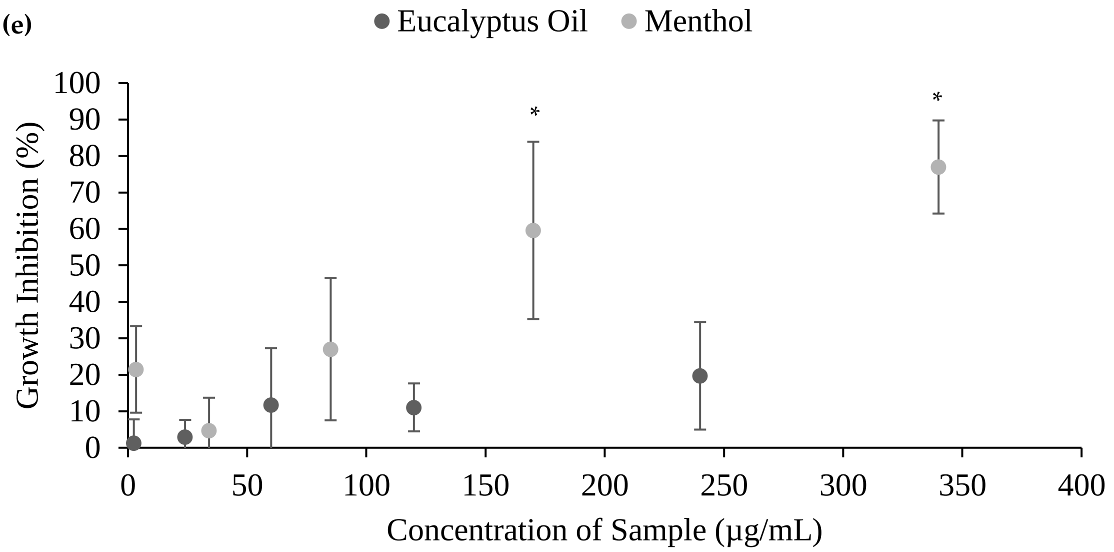

Supplementary Figure S2. Bacterial activity of eucalyptus oil (2.4–240 μg/mL) and menthol (3.4–340 μg/ml) with **(a)** *E. coli*, **(b)** *M. luteus*, **(c)** *B. subtilis*, **(d)** *S. epidermidis*, and **(e)** *L. acidophilus*. Points represent means ± standard deviation. * Indicates significant difference from negative control.

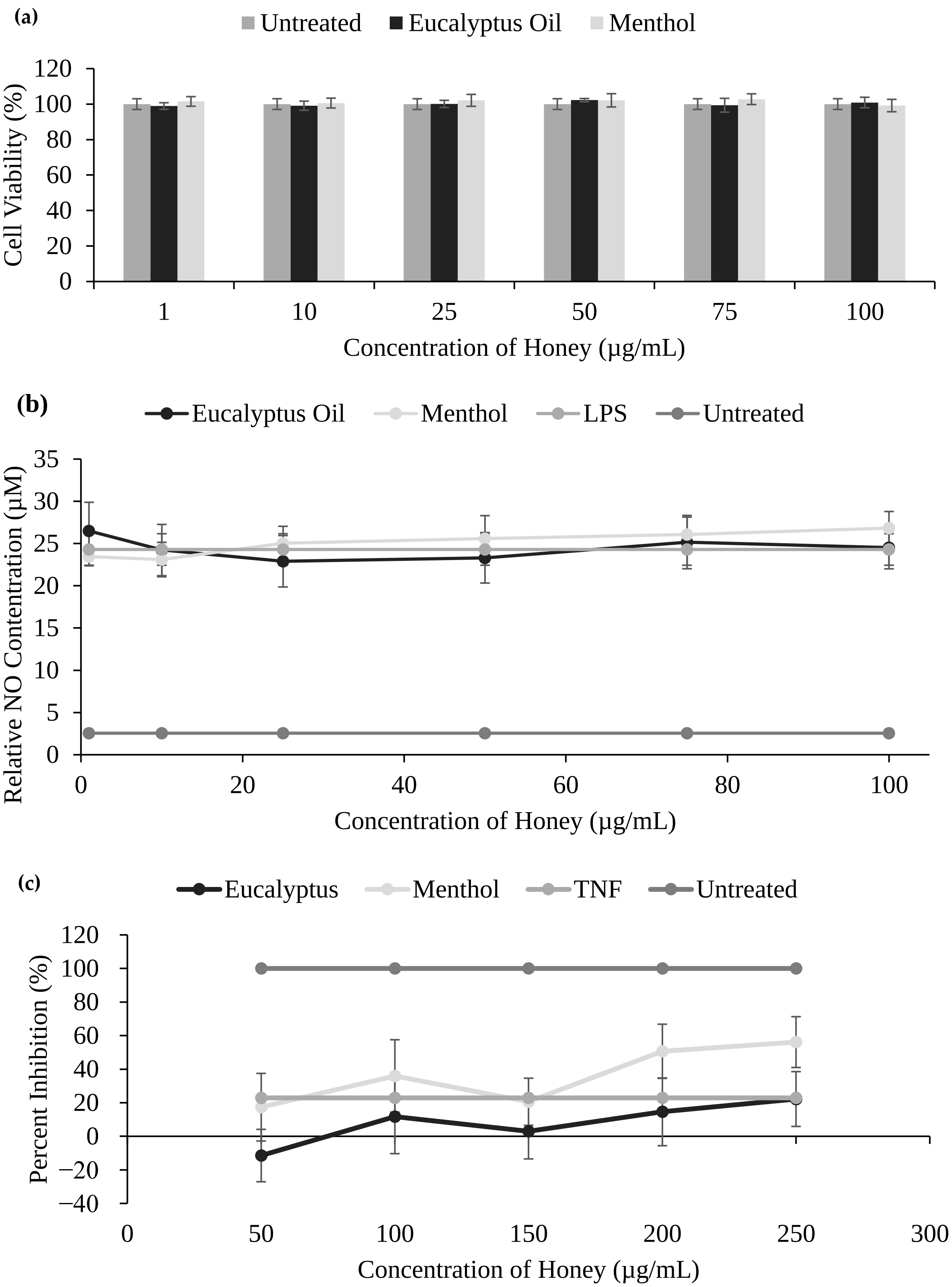

Supplementary Figure S3. *In vitro* anti−inflammatory assays of eucalyptus oil and menthol controls. **(a)** Cell viability of Raw 264.7 macrophages treated with eucalyptus and menthol (0.24% and 0.34% of honey concentration respectively). **(b)** Nitric oxide inhibition assay. Measures the ability of eucalyptus and menthol (0.24% and 0.34% of honey concentration respectively) to inhibit the production of nitric oxide by Raw 264.7 macrophages that are stimulated by LPS. The Untreated sample indicates cells that were only treated with PBS and not LPS, while the LPS

samples indicates cells that were treated with both PBS and LPS. **(c)** TNF−α cytotoxicity assay. This assay determines the ability of eucalyptus and menthol (0.24% and 0.34% of honey concentration respectively) to prevent loss of HC11 cell viability caused by TNF−α (1 ng/mL). The Untreated sample indicates cells that were only treated with PBS, while the TNF samples indicates cells that were treated with both PBS and TNF−α.

Supplementary Table S1. Table of Values−Activity of honey samples and controls with *E. coli, B. subtilis, M. luteus, S. epidermidis,* and *L. acidophilus*.

	Concen−tration of Honey (mg/mL)	Artificial Honey	Raw Honey	Honey Lozenge	Honey Drop	Eucalyptus Oil	Menthol
E. coli	1	−12.6 ± 20.8	21.0 ± 8.5	39.8 ± 3.2	31.3 ± 9.8	−5.5 ± 6.0	−1.2 ± 3.9
	10	10.6 ± 12.6	55.8 ± 5.9	64.2 ± 18.6	61.4 ±11.3	1.2 ± 11.3	2.5 ± 9.4
	25	12.7 ± 12.1	65.3 ± 13.4	45.2 ± 6.4	44.8 ± 3.7	2.4 ± 9.0	4.1 ± 3.9
	50	5.3 ± 12.4	54.4 ± 10.0	56.6 ± 11.9	44.8 ± 8.4	8.4 ± 3.3	9.5 ± 5.7
	100	−1.3 ± 4.6	63.5 ± 17.4	64.7 ± 13.2	43.9 ± 22.0	13.7 ± 10.0	14.8 ± 10.9
M. luteus	1	−0.1 ± 15.6	−27.1 ± 4.2	−28.4 ± 6.7	−29.8 ± 3.5	−0.6 ± 7.5	0.6 ± 6.2
	10	−23.2 ± 7.6	65.0 ± 16.0	69.2 ± 4.1	66.0 ± 4.0	2.7 ± 11.8	11.1 ± 4.8
	25	5.2 ± 19.0	63.9 ± 5.4	61.3 ± 5.7	58.2 ±12.5	1.9 ± 9.9	12.0 ± 4.1
	50	−7.8 ± 13.1	55.3 ± 9.4	46.7 ± 11.4	49.9 ± 18.2	1.0 ± 5.9	19.7 ± 7.5
	100	−20.0 ± 8.4	49.0 ± 11.9	26.1 ± 14.3	30.8 ± 6.2	2.7 ± 5.0	20.9 ± 14.1
B. subtilis	1	17.9 ± 11.7	5.0 ± 5.2	8.9 ± 11.4	6.4 ± 10.1	−0.3 ± 6.7	2.9 ± 1.4
	10	−19.2 ± 2.8	−19.4 ± 12.7	−2.7 ± 4.5	−11.3 ± 9.6	0.3 ± 2.4	−1.1 ± 3.5
	25	−8.3 ± 6.2	−5.0 ± 18.2	−27.9 ± 18.6	−23.6 ± 9.9	−8.8 ± 3.9	−7.6 ± 1.9
	50	−7.8 ± 11.8	−29.0 ± 14.5	−19.0 ± 7.5	−34.5 ± 20.3	−3.9 ± 4.5	−22.6 ± 0.7
	100	−10.0 ± 17.4	−32.5 ± 15.6	−25.7 ± 8.4	−47.6 ± 21.4	1.9 ± 2.4	−16.9 ± 2.0
S. epidermidis	1	−46.1 ± 10.5	−67.9 ± 3.2	−59.6 ± 3.0	−33.9 ± 22.6	−9.0 ± 7.9	−13.0 ± 7.5
	10	−26.6 ± 12.8	−57.4 ± 27.2	−46.7 ± 4.9	−45.7 ± 20.8	−0.4 ± 19.3	3.0 ± 15.8
	25	−23.1 ± 4.4	−56.4 ± 17.2	−59.4 ± 3.3	−65.7 ± 24.7	17.4 ± 20.1	30.2 ± 17.4
	50	−8.3 ± 8.2	−59.2 ± 4.7	−72.2 ± 18.8	−95.6 ± 14.3	52.1 ± 12.1	56.2 ± 14.6
	100	−4.1 ± 12.0	−66.5 ± 13.1	−46.4 ± 7.1	−43.4 ± 12.4	51.6 ± 22.7	56.4 ± 14.3
L. acidophilus	1	−10.1 ± 1.8	−24.9 ± 24.0	−33.0 ± 12.8	−7.3 ± 3.7	1.3 ± 6.5	21.5 ± 11.9
	10	22.9 ± 1.7	−29.1 ± 25.5	−31.6 ± 27.0	−22.0 ± 27.3	2.9 ± 4.7	4.7 ± 9.0
	25	16.6 ± 9.4	−18.3 ± 20.2	−18.3 ± 28.1	−27.3 ± 21.9	11.7 ± 15.6	27.0 ± 19.5
	50	31.9 ± 9.2	−19.3 ± 21.9	−5.9 ± 24.7	−25.1 ± 20.9	11.1 ± 6.6	59.6 ± 24.3
	100	38.4 ± 11.2	0.8 ± 26.6	25.9 ± 4.4	−4.6 ± 14.0	19.7 ± 14.7	77.0 ± 12.8

Supplementary Table S2. Nitric oxide production by Raw 264.7 cells (μM) when exposed to honey samples (1–100 μg/mL) and controls.

	Concentration of Nitric Oxide (μM)				
**Concentration of Honey (μg/mL)**	**Raw Honey**	**Honey Lozenge**	**Honey Drop**	**Eucalyptus Oil**	**Menthol**
1	25.09 ± 2.22	23.68 ± 2.25	23.00 ± 2.65	26.49 ± 3.39	23.44 ± 1.08
10	22.39 ± 2.74	19.63 ± 2.90	21.04 ± 3.41	24.23 ± 3.03	23.10 ± 2.04
25	23.74 ± 3.19	18.88 ± 2.30	20.50 ± 3.22	22.90 ± 3.04	25.04 ± 2.00
50	21.36 ± 2.70	20.44 ± 3.11	17.20 ± 2.24	23.31 ± 2.99	25.58 ± 2.72
75	20.70 ± 2.40	19.96 ± 3.17	16.16 ± 2.20	25.17 ± 3.16	26.08 ± 2.05
100	21.79 ± 2.53	20.22 ± 2.75	13.02 ± 2.90	24.53 ± 2.52	26.83 ± 1.96

LPS treatment = 24.30 ± 1.86; No treatment = 2.55 ± 0.24.

Supplementary Table S3. Percent inhibition of TNF−α cytotoxicity in HC11 cells by honey samples (50–250 µg/mL) and controls.

	Inhibition of TNF−α Cytotoxicity (%)				
**Concentration of Honey (µg/mL)**	**Raw Honey**	**Honey Lozenge**	**Honey Drop**	**Eucalyptus Oil**	**Menthol**
50	-	14.46 ± 15.77	15.28 ± 17.90	-	17.35 ± 20.14
100	62.12 ± 18.51	50.90 ±18.30	47.09 ± 16.21	11.71 ± 22.05	35.95 ± 21.57
150	85.13 ± 24.94	91.74 ± 22.85	55.74 ± 13.37	2.98 ± 16.45	20.58 ± 14.03
200	84.44 ± 19.31	95.92 ± 22.73	86.13 ± 15.07	14.58 ± 20.18	50.65 ± 16.14
250	101.98 ± 23.41	102.21 ± 13.54	96.08 ± 17.68	22.21 ± 16.33	56.13 ± 15.14

TNF treatment = 22.89 ± 7.77; (-) indicates no inhibition of TNF−α cytotoxicity.
